# Supplementary material for: Improvement of PBAT Processability and Mechanical Performance by Blending with Pine Resin Derivatives for Injection Moulding Rigid Packaging with Enhanced Hydrophobicity
Source: Polymers (Basel). 2020 Dec 2;12(12):2891. doi: 10.3390/polym12122891 (PMC7761566; doi:10.3390/polym12122891)
Supplement: Supplementary file 1 [file polymers-12-02891-s001.pdf]

Improvement of PBAT Processability and Mechanical Performance by Blending with Pine Resin Derivatives for Injection Moulding Rigid Packaging with Enhanced Hydrophobicity

Cristina Pavon [1,*], Miguel Aldas [1,2], Harrison de la Rosa-Ramírez[1], Juan López-Martínez [1], Marina P. Arrieta [3,4,*]

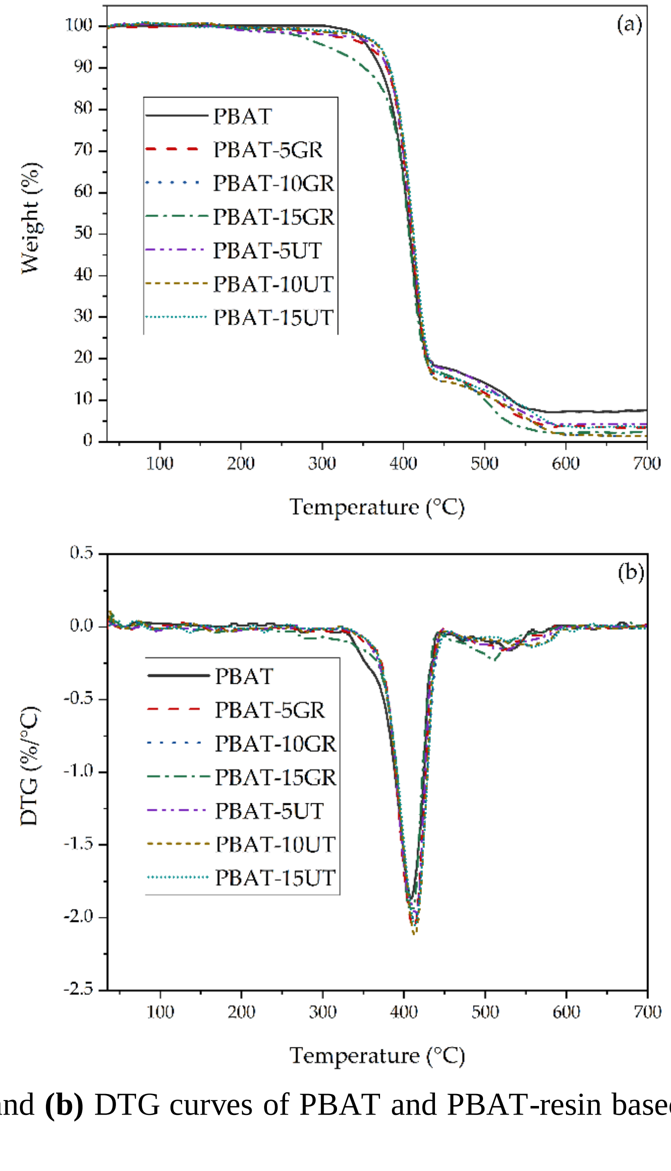

Figure S1. (a) TGA and **(b)** DTG curves of PBAT and PBAT-resin based formulations in 5, 10 and 15 wt.%
